# ﻿*Melanoserispenghuana* (Lactucinae, Cichorieae, Asteraceae), a new species from North-central Yunnan, China

**DOI:** 10.3897/phytokeys.238.116343

**Published:** 2024-02-01

**Authors:** Jia-Ju Xu, Ze-Huan Wang, Hong-Jin Dong, Qin Tian, Li Chen, Qian-Qian Zhong

**Affiliations:** 1 Department of Traditional Chinese Medicine Resources and Development, College of Pharmacy, Guizhou University of Traditional Chinese Medicine, Guiyang 550025, Guizhou, China Guizhou University of Traditional Chinese Medicine Guiyang China; 2 College of Biology and Agricultural Resources, Huanggang Normal University, Huanggang 438000, Hubei, China Huanggang Normal University Huanggang China; 3 Honghe Institute of Tropical Agriculture Science, Hekou 661300, Yunnan, China Honghe Institute of Tropical Agriculture Science Hekou China; 4 CAS Key Laboratory for Plant Diversity and Biogeography of East Asia, Kunming Institute of Botany, Chinese Academy of Sciences, Kunming 650201, Yunnan, China Kunming Institute of Botany, Chinese Academy of Sciences Kunming China

**Keywords:** *Melanoserislikiangensis*, morphology, Mt. Jiaozi Xueshan, new taxon, taxonomy

## Abstract

In this paper, we describe a new species, *Melanoserispenghuana*, from Mt. Jiaozi Xueshan located in North-central Yunnan, China. Despite its morphological similarities to *M.likiangensis*, *M.penghuana* exhibits distinct differences in leaf texture, shape of terminal lobes, indumentum of leaves, peduncles, and involucres, as well as the length of the achenes. Additionally, the conservation status of this species is classified as Vulnerable through data analysis from two field surveys.

## ﻿Introduction

The genus *Melanoseris* Decne. was initially established with only two species (Decaisne 1843). Later, Edgeworth (1846) expanded the genus to include seven species. However, the genus name remained unused by subsequent taxonomists for more than 165 years until Kilian reinstated its usage during the compilation of the Flora of China (Shih and Kilian 2011). As a result, numerous species within this genus were reassigned to other genera, such as *Lactuca* L., *Cicerbita* Wallr., *Prenanthes* L., *Cephalorrhynchus* Boiss., *Mulgedium* Cass., *Chaetoseris* Shih, and *Stenoseris* Shih, during this period of absence (Shih 1991, 1997; Zhu 2004; Zhu et al. 2004, 2006; Bano 2009; Bano and Qaiser 2009, 2010; Deng et al. 2011), making the delineation of species within this genus a challenging task. With the continuous in-depth research by taxonomists in recent years (Zhu 2004; Zhu et al. 2004, 2006; Kilian et al. 2009, 2017; Wang et al. 2009; Deng et al. 2011; Shih and Kilian 2011; Zhang et al. 2011; Wang et al. 2013, 2015, 2020; Abid et al. 2017; Ghafoor et al. 2017; Yin et al. 2018; Zhong et al. 2023), the species range of this genus has been gradually clarified. Currently, *Melanoseris* is the largest genus in the subtribe Lactucinae occurring in China, with a total of 20 species mainly distributed in the Pan-Himalayan region (Wang et al. 2013, 2015, 2020; Yin et al. 2018; Zhong et al. 2023).

During a field survey of Mt. Jiaozi Xueshan in 2021, we discovered a species of *Melanoseris* growing on the steep slopes on both sides of the Jiulonggou valley. It had large leaves, and the terminal lobes of leaves were extremely elongated, which caught our attention. Subsequent in-depth research confirmed that it may be an unpublished species of *Melanoseris*. To test the stability of its elongated terminal lobes, we conducted another field investigation in 2022 to examine its plant’s morphological variation and population size. The results of the investigation showed that the unique terminal lobes were a stable characteristic within the population. Further morphological studies and analysis revealed both similarities and distinct differences between this plant and *M.likiangensis* (Franchet) N.Kilian & Ze H.Wang. Based on these findings, the authors reached the conclusion that this plant represents a newly identified species, which is comprehensively described and illustrated in this study.

## ﻿Material and methods

To conduct the morphological description of the new species, we observed and photographed live plants in the field. Additionally, we utilized herbarium collections (KUN, GTZM) from these occasions. For morphological comparative analysis, we referred to the keys of the genus and descriptions of the species in Flora Reipublicae Popularis Sinicae (Shih 1997) and Flora of China (Shih and Kilian 2011). To facilitate further comparisons, we examined the protologue of *M.likiangensis* (Franchet 1895), as well as the specimen photographs in the herbaria IBSC, E, K, KUN, P, and PE. The morphology of trichomes and pappus, as well as the length of ligules, anther tubes, and achenes, were observed or measured using an anatomy microscope (OD500H) or a light microscope (Olympus DP72) on fresh or dried specimens. The classification of trichomes in this study followed Ramayya’s classic treatment of trichomes on Compositae (Ramayya, 1962). Photographs were taken using a Canon EOS 77D and a Dell E2014Hf camera. Figures were edited, arranged, and merged using Adobe Illustrator CS4. Additionally, a distribution map was generated with QGIS 3.32.2.

## ﻿Results

### ﻿Taxonomy

#### ﻿ Melanoseris penghuana

Taxon classificationPlantaeAsteralesAsteraceae

Ze H.Wang & H.J.Dong sp. nov.

DFAD7429-8E37-5382-8830-3E653C00EB2B

urn:lsid:ipni.org:names:77335477-1

Figs 1 , 2

##### Type.

China, Yunnan Province, Kunming City, Dongchuan District, Mt. Jiaozi Xueshan, Jiulonggou, 26°09.95'N, 102°54.83'E, alt. 3269 m, 12 Oct 2022, *Tian Qin et al.* 20221001 (holotype: KUN1584358!, isotypes: KUN1584356, 1584357!, GTZM0220112, 0220113!).

##### Diagnosis.

*Melanoserispenghuana* is most similar to *M.likiangensis*, but differs from the latter primarily in the following characteristics: leaves thick papery (vs. papery), clearly hairy (vs. glabrous or sparsely hairy), terminal lobes of basal and lower leaves elongated triangular (vs. broad triangular), the length 3–4 times (vs. 1–1.5 times) that of the width; peduncles covered with simple filiform hairs (vs. multiseriate capitate glandular hairs), involucres glabrous (vs. glandular hispid), achenes ca. 9.5 mm (vs. ca. 7 mm).

**Figure 1.**
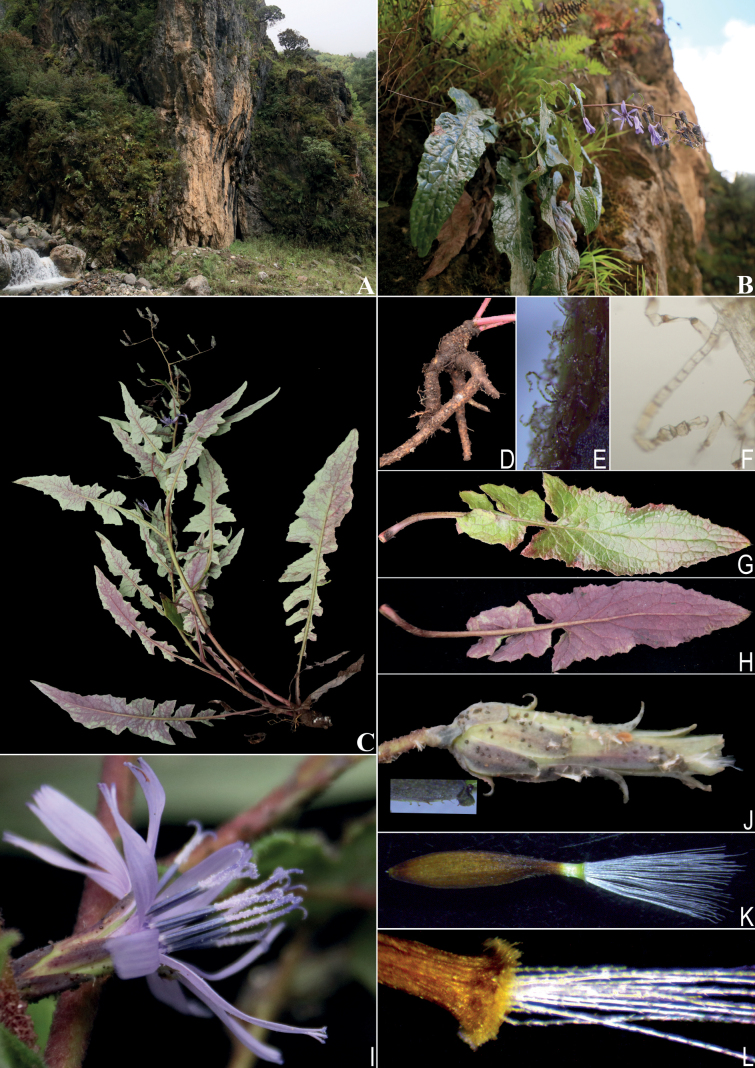
*Melanoserispenghuana* sp. nov. **A** habitat **B**, **C** plants **D** root **E** trichomes on the peduncle under an anatomy microscope **F** trichomes on the peduncle under a light microscope (simple filiform hairs) **G, H** basal leaves **I** capitulum **J** involucre **K** achene **L** pappus. Photographed by Qin Tian.

##### Description.

Perennial herbs, 30–55 cm tall. Roots fleshy 0.5–1.5 cm in diameter, cylindrical, branched. Stems 1–2, emerging from the apex of a rhizome, erect, with a basal diameter of 3–4 mm, apically branched, covered with simple filiform hairs, especially at the apex. Basal leaves 24–40 × 4–9 cm, thick papery, elongated and narrowly elliptic, lyrately pinnatipartite; terminal lobes 10–18 × 4–8 cm, elongated triangular, apical acuminate to long acuminate; lateral lobes 2–5 pairs, 0.7–5 × 0.4–3 cm, inverted triangular, widest at base. Petioles 2–9 cm long, sparsely pubescent. Lower and middle stem leaves 11–23 × 3–5 cm, like basal leaves but smaller, lyrately pinnatipartite; terminal lobes 5–13 × 2–4 cm, elongated triangular, apex long acuminate; lateral lobes 3–4 pairs, 0.5–3 × 0.3–2 cm, inverted triangular. Petioles 1–2 cm long, narrowly winged, basally widened and clasping. Upper stem leaves like middle stem leaves but smaller, with a shorter petiole conspicuous winged and auriculately clasping. Uppermost leaves lanceolate, less divided or entire. All the leaves clearly covered with simple filiform hairs on both surfaces, especially on the veins; margins coarsely dentate, green adaxially, usually purplish-red abaxially. Capitulescence racemiform to narrowly paniculiform; peduncles clearly covered with simple filiform hairs, bracts few, inconspicuous, scale-like. Capitula few, pendulous, with 10–12 florets. Involucre 1.6–1.9 × 0.5 cm, cylindrical, dark purplish green, glabrous. Phyllaries imbricate, 4–5-seriate, with apex acute to acuminate, conspicuously reversed; outer phyllaries 2–3 × 1 mm, triangular ovate, margin occasionally with a few transparent protrusions; middle phyllaries 7–10 × 2–3 mm, long ovate; innermost phyllaries 8, 16–19 × 2 mm, narrowly lanceolate. Florets ligulate, tube ca. 4 mm long, light purple, ligules ca. 12 × 1.5 mm, 5-toothed at the apex, purple. Stamens synantherous, anther tube 5.0–5.2 mm long, dark purple. Ovary inferior, flattened, ellipsoid, style ca. 16 mm long, apically bifid, stigmatic braches ca. 1.2 mm long, long and acuminate, evenly coated with elongate collection hairs. Achenes 10 × 2 mm, fusiform, dark brown, each side with 3 raised longitudinal ribs, surface sparsely hairy, and apex contracted into a 3 mm beak, beak discolorous, with the top half being white. Pappus 2-seriate, white, outer seriate 0.1–0.2 mm, inner seriate ca. 7 mm long, finely serrated.

**Figure 2.**
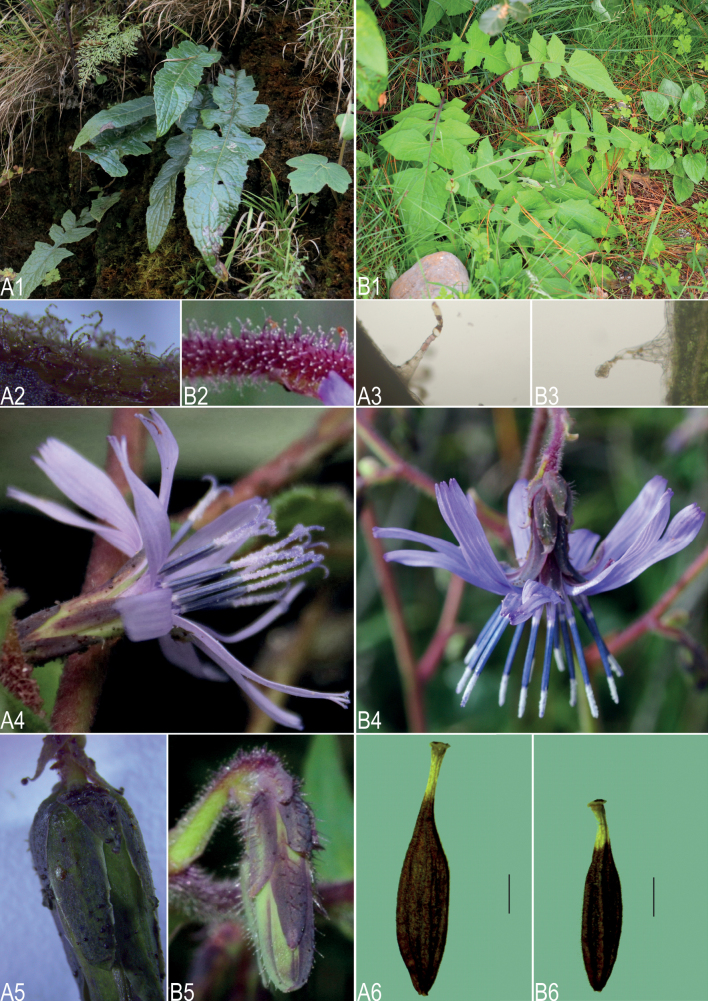
A morphological comparison between *Melanoserispenghuana* and *M.likiangensis***A1–A6***M.penghuana*: **A1** basal leaves **A2** trichomes on the peduncle under an anatomy microscope **A3** simple filiform hairs on the leaves under a light microscope **A4** capitulum **A5** involucre **A6** achene **B1–B6***M.likiangensis*: **B1** basal leaves **B2** trichomes on the peduncle under an anatomy microscope **B3** multiseriate capitate glandular hairs on the leaves under a light microscope **B4** capitulum **B5** involucre **B6** achene. Scale bars: 2 mm. **A1–A2, A4, A5** were photographed by Qin Tian, others were photographed by Ze-Huan Wang.

##### Distribution and habitat.

**Figure 3.**
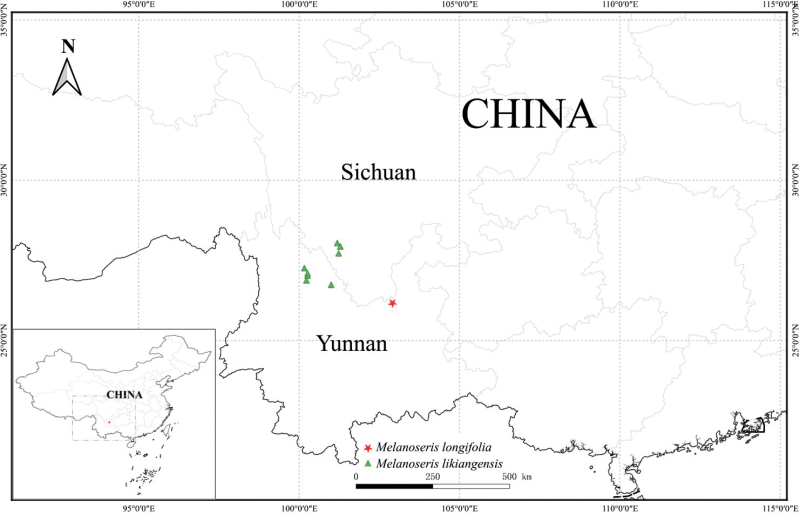
Distribution map of *Melanoserispenghuana* and *M.likiangensis*.

*Melanoserispenghuana* is currently observed growing on steep grassy slopes along the valley edge of Jiulonggou, Mt. Jiaozi Xueshan, at an elevation of approximately 3200 m (Fig. 3). The companion plants mainly include *Youngiamairei* (H.Léveillé) Babcock et Stebbins (Asteraceae), *Saxifragafilicaulis* Wallich ex Seringe (Saxifragaceae), *Silenedelavayi* Franchet (Caryophyllaceae), *Rubusdelavayi* Franchet (Rosaceae), *Liliumsempervivoideum* H.Léveillé (Liliaceae), *Oreocharismairei* H.Léveillé (Gesneriaceae) etc.

##### Phenology.

Flowering and fruiting from September to October.

##### Etymology.

The specific epithet “*penghuana*” is named in honor of Professor Hua Peng, a renowned expert in plant taxonomy in China, for his outstanding contributions to the protection of Mt. Jiaozi Xueshan.

##### Vernacular name.

Simplified Chinese:彭氏毛鳞菊; Chinese Pinyin: Péngshì Máolínjú.

##### Conservation status.

*Melanoserispenghuana* is found distributed along the steep grassy slopes on both sides of the Jiulonggou valley in Mt. Jiaozi Xueshan, Yunnan Province. In 2021 and 2022, the authors discovered three subpopulations; each of them had a considerable number of flowering plants and seedlings with only rosette leaves. Preliminary estimates suggest that there are more than 250 mature individuals. Although the current survey indicates that the distribution of *M.penghuana* is relatively concentrated, it is worth noting that its distribution areas are located within the Jiaozi Xueshan National Nature Reserve, where human disturbance is minimal. As a result, its habitat is relatively well protected. Thus, based on its very restricted population and the number of mature individuals estimated to be larger than 250 but fewer than 1000 (IUCN 2012, 2022), this new species should be classified as Vulnerable (VU; criteria D1).

##### Additional specimens examined.

China, Yunnan Province, Kunming City, Dongchuan District, Mt. Jiaozi Xueshan, Jiulonggou, 26°9.97'N, 102°54.92'E, alt. 3279 m, 6 Oct 2021, Dong Hong-Jin et al. D634 (KUN1584359!, GTZM0220114!); ibid, 26°09.95'N, 102°54.87'E, alt. 3281 m, 12 Oct 2022, Tian Qin et al. 20221002 (KUN1584360!, GTZM0220115!).

## ﻿Discussion

*Melanoserislikiangensis* is an endemic species found in Northwest Yunnan, China (Fig. 3). Most of its specimens were collected several decades ago. Currently, there are still unresolved issues regarding the classification of *M.likiangensis*. For example, among the specimens defined as *M.likiangensis*, there are two types of inner involucral bracts: some have 5 bracts while others have 8 bracts. Furthermore, there is no consensus yet on whether *M.bonatii* (Beauverd) Ze H.Wang, a species found in Northeastern Yunnan, is conspecific with *M.likiangensis*. Resolving these taxonomical issues necessitates more specimen studies and specialized field investigations. To accurately compare the morphological characteristics of *M.penghuana* and *M.likiangensis*, we consulted the original description of *M.likiangensis* in the protologue (Franchet 1895). The main morphological differences between these two species are detailed in Table 1.

**Table 1.** Comparison of the morphological characteristics between *Melanoserispenghuana* and *M.likiangensis*.

Characteristics	*M.penghuana*	*M.likiangensis*
**Leaf texture**	thick papery	papery
**Terminal lobes of basal and lower leaves**	elongated triangular, ca. 7–19 cm, the length is 3–4 times that of the width, apical acuminate to long acuminate	broad triangular, ca. 7 cm, the length is 1–1.5 times that of the width, apical acuminate to acute
**Leaves trichomes**	all the leaves clearly covered with simple filiform hairs on both surfaces, especially on the abaxially veins	all the leaves typically glabrous on both surfaces, with the occasional presence of sparsely distributed multiseriate capitate glandular hairs adaxially
**Peduncles trichomes**	simple filiform hairs	multiseriate capitate glandular hairs
**Involucres**	glabrous, margin occasionally with a few transparent protrusions	the middle vein of outer and middle phyllaries has one row of multiseriate capitate glandular hairs outside
**Achenes**	ca. 9.5 mm	ca. 7 mm

The continuity of a new species’ population often receives significant attention from taxonomists. In the case of *Melanoserispenghuana*, the expansion of its population is influenced by a combination of unfavorable and favorable factors. One of the unfavorable factors is the relatively low number of head inflorescences on each plant, and what’s worse, there are only 10–12 florets per inflorescence. Furthermore, the author observed that the inflorescences of this species, similar to *Sinoserismuliensis* (Y.S.Chen, L.S.Xu & R.Ke) Ze H.Wang, N.Kilian & H.Peng (Wang et al. 2020) and *M.kangdingensis* Ze H.Wang (Zhong et al. 2023), are susceptible to parasitism by certain insects. These factors contribute to a lower quantity of seeds produced by *M.penghuana* plants.

On the other hand, there are several favorable factors contributing to the expansion of the *Melanoserispenghuana* population. Firstly, the species is distributed in the Jiaozi Xueshan National Nature Reserve, where the habitat is relatively well-protected. Secondly, the recent relocation of residents from Jiulonggou Village has reduced human disturbance in the area where *M.penghuana* grows. Thirdly, based on the field survey conducted by the authors in 2022, the population of *M.penghuana* is relatively large and not as endangered as many other recently discovered plants (Ma et al. 2019; Huang et al. 2020; Qiu et al. 2020; Nong et al. 2021). Lastly, compared with the recently published *M.kangdingensis* (Zhong et al. 2023), the growing environment of *M.penghuana* is also steep, but the soil layer of its habitat is relatively well-developed. Therefore, the probability of successful seed germination of *M.penghuana* after landing is much higher than that of *M.kangdingensis*.

## Supplementary Material

XML Treatment for Melanoseris penghuana

## ﻿Additional information

### Author ORCIDs

Jia-Ju Xu https://orcid.org/0009-0004-2852-0611

Ze-Huan Wang https://orcid.org/0000-0002-7369-5005

Hong-Jin Dong https://orcid.org/0000-0002-7098-9374

Qin Tian https://orcid.org/0000-0002-3025-8948

Li Chen https://orcid.org/0000-0001-6030-2439

Qian-Qian Zhong https://orcid.org/0009-0002-7051-1503

### Data availability

All of the data that support the findings of this study are available in the main text.
